# Clinical impact of high platelet reactivity in patients with atrial fibrillation and concomitant percutaneous coronary intervention on dual or triple antithrombotic therapy

**DOI:** 10.1007/s11239-023-02784-z

**Published:** 2023-03-11

**Authors:** M. Berteotti, A. M. Gori, B. Giusti, A. Fortini, G. Grossi, N. Ciardetti, A. Migliorini, E. Lotti, R. Valenti, C. Di Mario, N. Marchionni, R. Marcucci

**Affiliations:** 1 Department of Experimental and Clinical Medicine, University of Florence, Largo Brambilla, 3, 50134 Florence, Italy; 2 Division of Interventional Cardiology, Cardiothoracovascular Department, Careggi University Hospital, Florence, Italy; 3 Thrombosis Center, Careggi University Hospital, Florence, Italy

**Keywords:** Platelet reactivity, Triple antithrombotic therapy, CYP2C19 polymorphism, Antiplatelet therapy, Oral anticoagulant

## Abstract

High platelet reactivity (HPR) on clopidogrel is an established thrombotic risk factor after percutaneous coronary intervention (PCI). The introduction of more potent antiplatelet drugs has partially surpassed this issue. However, in the setting of concomitant atrial fibrillation (AF) and PCI clopidogrel is still the most adopted P2Y_12_ inhibitor. In the present study all consecutive patients with history of AF discharged from our cardiology ward with dual (DAT) or triple (TAT) antithrombotic therapy after a PCI from April 2018 to March 2021 were enrolled in an observational registry. For all subjects, blood serum samples were collected and tested for platelet reactivity by arachidonic acid and ADP (VerifyNow system) and genotyping of the CYP2C19*2 loss-of-function polymorphism. We recorded at 3 and 12-months follow-up: (1) major adverse cardiac and cerebrovascular events (MACCE), (2) major hemorrhagic or clinically relevant non-major bleeding and (3) all-cause mortality. A total of 147 patients were included (91, 62% on TAT). In 93.4% of patients, clopidogrel was chosen as P2Y_12_ inhibitor. P2Y_12_ dependent HPR resulted an independent predictor of MACCE both at 3 and 12 months (HR 2.93, 95% C.I. 1.03 to 7.56, p = 0.027 and HR 1.67, 95% C.I. 1.20 to 2.34, p = 0.003, respectively). At 3-months follow-up the presence of CYP2C19*2 polymorphism was independently associated with MACCE (HR 5.21, 95% C.I. 1.03 to 26.28, p = 0.045). In conclusion, in a real-world unselected population on TAT or DAT, the entity of platelet inhibition on P2Y_12_ inhibitor is a potent predictor of thrombotic risk, suggesting the clinical utility of this laboratory evaluation for a tailored antithrombotic therapy in this high-risk clinical scenario.

**Graphical abstract:**

The present analysis was performed in patients with AF undergoing PCI on dual or triple antithrombotic therapy. At 1 year follow-up MACCE incidence was consistent, and it was not different in different antithrombotic pattern groups. P2Y_12_ dependent HPR was a potent independent predictor of MACCE both at 3- and 12-months follow-up. In the first 3 months after stenting the carriage of CYP2C19*2 allele was similarly associated with MACCE. Abbreviation: DAT, dual antithrombotic therapy; HPR, high platelet reactivity; MACCE, major adverse cardiac and cerebrovascular events; PRU, P2Y_12_ reactive unit; TAT, triple antithrombotic therapy. Created with BioRender.com.

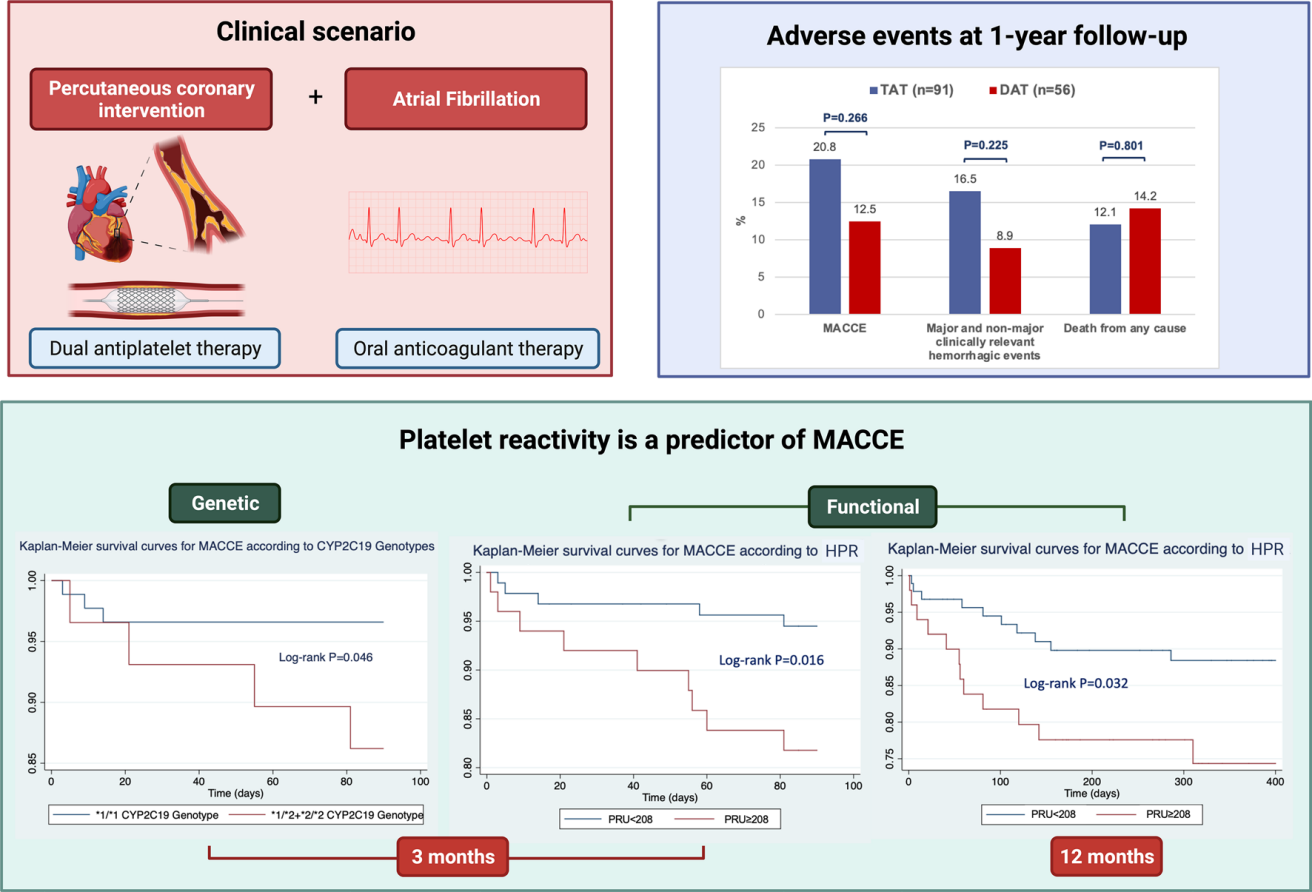

**Supplementary Information:**

The online version contains supplementary material available at 10.1007/s11239-023-02784-z.

## Highlights

- Despite the large body of evidence confirming the role of residual platelet reactivity after PCI, less is known in the setting con combined antiplatelet and anticoagulant therapy.
- In a real-world unselected population of patients with AF undergoing PCI mainly after acute coronary syndrome (ACS), discharged in dual or triple antithrombotic therapy, P2Y_12_ dependent HPR assessed by VerifyNow and the carriage of CYP2C19*2 loss-of-function polymorphism independently predict the incidence of MACCE at 1-year follow-up.
- In the setting of combined antithrombotic therapy, both functional and genetic platelet function test have a pivotal role in guiding antithrombotic strategy, since we are often dealing with ACS patients receiving mostly clopidogrel alone or double antiplatelet therapy for a brief amount of time.

## Introduction

The optimal antithrombotic treatment regimen for patients with atrial fibrillation (AF) undergoing percutaneous coronary intervention (PCI) is a clinical dilemma [1]. Although dual antiplatelet therapy (DAPT) with aspirin and a P2Y_12_ inhibitor is effective in reducing cardiac events and stent thrombosis after PCI [2, 3], oral anticoagulation is the therapy of choice for the prevention of stroke and systemic embolism in patients with AF [4]. However, triple antithrombotic therapy (TAT) is known to be associated with an excess in major bleeding, ranging from 4 to 12% within the first year of treatment [5, 6] and hemorrhagic risk is 4 times higher than that observed with aspirin alone [7]. The occurrence of a bleeding complication is known to affect the prognosis of the patients, as it determines the discontinuation of all antithrombotic drugs [8]. As demonstrated in recent trials, this risk is mitigated when direct oral anticoagulants (DOAC) are prescribed instead of vitamin K antagonists (VKA) and aspirin is discontinued (dual antithrombotic therapy, DAT) [9–13]. For this reason, both American and European latest guidelines agree that aspirin should be maintained for 1 to 7 days (or until hospital discharge), with the option to prolong up to 30 days if the thrombotic or ischemic risk is high and the bleeding risk is low [14, 15]. However, a meta-analysis encompassing 10 234 patients (DAT = 5496 vs. TAT = 4738) demonstrated that despite the significant reduction in ISTH major or clinically relevant non-major bleeding with DAT compared with TAT, this benefit was counterbalanced by a significant increase of stent thrombosis (RR 1.59, 95% CI 1.01 to 2.50; P = 0.04; I2 = 0%) and a trend towards higher risk of myocardial infarction (MI) [13]. The implementation of tools allowing for the identification and prediction of platelet inhibition has recently shown to improve outcomes [16]. Several papers documented the role of high platelet reactivity (HPR) on P2Y_12_ inhibitors and of the carriage of the *2 or *3 polymorphism of Cytochrome P450 2C19 (CYP2C19, involved in the bioactivation of clopidogrel) as independent predictors of ischemic events [17–20]. Nevertheless, these data were obtained in the clinical setting of acute coronary syndrome (ACS) patients undergoing PCI and discharged on clopidogrel as P2Y_12_ inhibitor (condition which has been partially surpassed by the more potent inhibitors) and in the absence of anticoagulants. Actually, scarce and unconclusive data have been provided so far in the setting of combined antithrombotic therapy, which has resurrected clopidogrel as P2Y_12_ inhibitor of choice.

The aim of the present study was therefore to evaluate the possible clinical relevance of genetic and platelet function testing (PFT) in a real-world population with concomitant AF and PCI on DAT or TAT.

## Methods

### Study population

The study population was enrolled in the observational prospective “Registry on the prescription of anticoagulant agents in association with double antiplatelet therapy in high cardiovascular risk patients with concomitant AF and PCI” (number of registry of ethics committee’s opinion: 12485_bio). The study started on April 2018 with the aim to identify possible laboratory predictors of bleeding and ischemic events in a real-world population. In the present analysis we included patients enrolled until March 2021 in the Cardiovascular department of the Careggi University hospital, Florence. Patients with a life expectancy lower than one year were excluded. Patients with the required characteristics, were informed about the study procedures and then signed the informed consent. Clinical and anamnestic data as well as general laboratory examinations performed during hospitalization were recorded. Information about index event such as coronary artery disease (CAD) presentation and procedural PCI aspects were also collected. For each patient CHA_2_DS_2_-VASc and HAS-BLED scores were calculated, and therapy at discharge was registered, including type of antiplatelet or anticoagulant agent. The choice of the therapy at discharge and at subsequent evaluations was blinded as clinicians were unaware of the results of the present analysis.

### Laboratory analyses

Blood samples were obtained for all subjects and the following analysis were performed: platelet function tests [TXA2 dependent platelet reactivity (ARU), P2Y_12_ dependent platelet reactivity (PRU)]; and genetic testing. Platelet reactivity was assessed by VerifyNow System. The cut-off values used for identification of patients undergoing PCI at higher risk of adverse cardiovascular events were 208 PRU and 550 ARU [21, 22]. Genomic DNA was isolated from venous peripheral blood using Tecan, Freedom EVO liquid handler (Tecan Group) and the magnetic bead based GeneCatcher gDNA Blood kit (Invitrogen). DNA purity and concentration were determined by NanoDrop spectrophotometer (Thermo Scientific). Genotyping of the CYP2C19*2 loss-of-function polymorphism (681G > A, rs4244285) was performed using TaqMan validated Drug Metabolism Genotyping assay with the 7900HT Sequence Detection System (Applied Biosystems).

### Follow-up and endpoints

Follow-up was performed at 1, 3, 6 and 12 months. The endpoints of the study were: (1) major cardiovascular and cerebrovascular events (MACCE: MI, stent thrombosis, percutaneous revascularization, ischemic stroke, cardiovascular death); (2) major and non-major clinically relevant hemorrhagic events, according to ISTH classification (International Society of Thrombosis and Haemostasis) [23]; and (3) all-cause mortality. MI was defined according to the fourth universal definition of 2018 ESC guidelines [24]. Ischemic stroke was defined as an episode of neurological dysfunction caused by focal cerebral, spinal, or retinal infarction persisting ≥ 24 h or until death [25]. At each step of follow-up, antithrombotic therapy modifications were recorded. Follow-up was interrupted after the occurrence of an adverse event, death, or because of explicit denial of patients to give information.

### Statistical analysis

Statistical analysis was performed with SPSS (Statistical Package for Social Sciences, Chicago, Illinois, USA), version 25.0. Categorical data were reported as frequencies. Continuous variables were reported as mean and standard deviation or median and interquartile range, as appropriate (Kolmogorov Smirnov test was performed). Dichotomic variables were compared through Chi square test or Fisher exact Test, while continuous variable by T student or Mann–Whitney. We calculated the risk of clinical endpoints at 3 months and 1-year follow-up. Cumulative survival curves were analysed with Kaplan–Meier method. The univariable and multivariable analyses to evaluate the independent contribution of clinical and laboratory variables to the endpoints were performed by Cox proportional hazards model. The variables that reached the highest significance at the univariable analysis (p < 0.1) were considered in the final multivariable model (according to backward logistic regression).

## Results

A total of 147 patients were included in the study; the mean age was 78 ± 8 years and 48 (33%) were women. The most common CAD manifestation was ACS (n = 103, 70%); in about 25% of patients, AF was diagnosed for the first time during index hospitalization. Four patients had a mechanical prosthetic valve. The antithrombotic therapy at discharge was DAT in 56 cases (38%) e TAT in 91 (62%). Patients on TAT had a significantly lower BMI, a more complex CAD in terms of number of diseased and treated vessels and a worse renal function; in particular, all patients in dialysis were discharged in TAT (Table 1). The anticoagulant of choice was a DOAC in 53 patients in TAT (58%) and 47 cases in DAT (84%). All patients in TAT but two received clopidogrel as P2Y_12_ inhibitor, while in the other cases ticagrelor was prescribed. Among patients on DAT, ticagrelor was prescribed in 6 patients (10.7%) and aspirin in one (0.7%). As concerns the anticoagulant therapy, a VKA in TAT was preferred in younger patients and in those with a previous stroke or transient ischemic attack; patients in this group had also a higher mean HAS-BLED score and a lower mean eGFR as compared to those in DOAC-TAT group. No statistic difference was observed for other variables among patients in DAT (Supplementary Table 1).

**Table 1:** Baseline characteristics of the study population according to antithrombotic pattern

	Total (n = 147)	TAT (n = 91)	DAT (n = 56)	P value
Age [yrs], (mean ± SD)	78 ± 8	77 ± 8	79 ± 8	0.093
Male sex, n (%)	99 (67.3)	62 (68.1)	37 (66.1)	0.796
Hypertension, n (%)	122 (83.0)	74 (81.3)	48 (85.7)	0.491
Dyslipidemia, n (%)	86 (58.5)	58 (63.7)	28 (50.0)	0.101
Smokers, n (%)	16 (10.9)	11 (12.1)	5 (8.9)	0.361
Former Smokers, n (%)	58 (39.4)	39 (42.9)	19 (33.9)	
Diabetes mellitus, n (%)	50 (34.0)	34 (37.4)	16 (28.6)	0.275
Family history of CVD, n (%)	22 (15.0)	15 (16.5)	7 (12.5)	0.511
BMI [kg/m^2^] (mean ± SD)	27.0 ± 4.0	26.3 ± 3.5	28.0 ± 4.5	0.014
Prior MI, n (%)	53 (36.0)	33 (36.3)	20 (35.7)	0.946
Prior PCI, n (%)	64 (43.5)	41 (45.1)	23 (41.1)	0.636
Prior CABG, n (%)	12 (8.2)	8 (8.8)	4 (7.1)	0.723
Prior TIA/stroke, n (%)	18 (12.2)	11 (12.1)	7 (12.5)	0.941
PAD, n (%)	48 (32.7)	28 (30.8)	20 (35.7)	0.589
Prior bleeding, n (%)	12 (8.2)	5 (5.5)	7 (12.5)	0.212
ACS, n (%)	103 (70.1)	68 (74.7)	35 (62.5)	0.116
UA, n (%)	27 (18.4)	22 (24.2)	5 (8.9)	0.141
NSTEMI, n (%)	55 (37.4)	33 (36.3)	22 (39.3)	
STEMI, n (%)	21 (14.3)	13 (14.3)	8 (14.3)	
MI, n (%)	66 (44.9)	46 (50.1)	20 (53.5)	0.048 (vs UA)
LVEF [%], (mean ± SD)	46 ± 11	45 ± 11	48 ± 11	0.087
Number of diseased vessels	2 (1–3)	2 (1–3)	1.5 (1–2)	< 0.001
1 n (%)	45 (30.6)	17 (18.9)	28 (50.0)	
2 n (%)	55 (37.4)	38 (42.2)	17 (30.4)	
3 n (%)	46 (31.3)	35 (38.9)	11 (19.6)	
LM disease, n (%)	41 (27.9)	32 (35.6)	9 (16.1)	0.011
Number of treated vessels, median (IQR)	1 (1–2)	1 (1–2)	1 (1–2)	0.020
Number of stent, median (IQR)	2 (1–3)	2 (1–3)	1 (1–2)	0.016
Total stent length, mm (mean ± SD)	49.2 ± 34.1	53.4 ± 3.7	41.4 ± 32.1	0.054
CHA_2_DS_2_-VASc score (mean ± SD)	4.7 ± 1.2	4.8 ± 1.4	4.7 ± 11.2	0.924
HAS-BLED score (mean ± SD)	2.4 ± 0.7	2.5 ± 0.7	2.3 ± 0.6	0.695
Statin, n (%)	128 (87.0)	82 (90.1)	46 (82.1)	0.207
Atorvastatin, n (%)	54 (69.4)	70 (76.9)	32 (57.1)	
Rosuvastatin, n (%)	16 (10.9)	9 (9.9)	7 (12.5)	
Pravastatin, n (%)	1 (0.7)	0 (0)	1 (1.8)	
Simvastatin, n (%)	9 (6.1)	3 (3.3)	6 (10.7)	
PPI, n (%)	136 (92.5)	85 (93.4)	51 (91.1)	0.749
Lansoprazol, n (%)	25 (17.0)	14 (15.4)	11 (19.6)	
Omeprazol, n (%)	3 (2.0)	2 (2.2)	1 (1.8)	
Pantoprazol, n (%)	108 (73.5)	69 (75.8)	39 (69.6)	
WBC [× 10^3/^uL] (mean ± SD)	7.91 ± 2.99	7.99 ± 3.30	7.80 ± 2.43	0.712
Hb [g/dL], (mean ± SD)	12.0 ± 2.5	11.8 ± 1.9	12.3 ± 3.3	0.224
Platelets [10^3^/uL], (mean ± SD)	221 ± 85	221 ± 87	222 ± 82	0.921
MCV [fL] (mean ± SD)	89.3 ± 8.1	89.1 ± 9.0	89.8 ± 6.3	0.597
Creatinin [mg/dL], (mean ± SD)	1.4 ± 0.9	1.6 ± 1.2	1.1 ± 0.3	0.001
eGFR [mL/min/1.73m^2^], (mean ± SD)	52 ± 23	48 ± 24	57 ± 22	0.023
Dialysis, n (%)	7 (4.7)	7 (10.6)	0	0.035

*BMI* body mass index; *CABG* coronary artery bypass grafting; *CVC* cardiovascular disease; *DTA* dual antithrombotic therapy; *eGFR* estimated glomerular filtration rate; *Hb* Hemoglobin; *IQR* interquartile range; *LVEF* left ventricular ejection fraction; *LM* left main; *MCV* mean corpuscular volume; *MI* myocardial infarction; *NSTEMI* non-ST elevation myocardial infarction; *PCI* percutaneous coronary intervention; *PPI* proton pump inhibitor; *SD* standard deviation; *STEMI* ST elevation myocardial infarction; *TAT* triple antithrombotic therapy; *TIA* transient ischemic attack; *UA* unstable angina; *WBC* white blood cell count

In the whole population median PRU value was 177 U (IQR 108 to 229) and median ARU value was 486 U (IQR 422 to 552). P2Y_12_-dependent HPR was observed in 50 patients (34%) and TXA2 HPR was present in 13 subjects discharged in TAT (14%). Concomitant P2Y_12_ and TXA2 HPR was found in 15 patient (10.2%). The genotyping was available for 115 subjects. The genotype distribution of the CYP2C19*2 polymorphism in the studied population was 0.9% homozygotes *2/*2, 24.3% heterozygotes *1/*2, 74.8% homozygotes *1/*1. The polymorphism was in Hardy–Weinberg equilibrium. The median PRU levels in patients receiving clopidogrel as P2Y_12_ inhibitor were 210 U (IQR 159 to 247 U) in *2/*2 and *1/*2 patients and 157 U (IQR 113 to 222 U) in *1/*1 (wild-type) patients, p = 0.039.

### Follow-up

Median follow-up was 401 days (IQR 241 to 588). In TAT subgroup, aspirin was interrupted after one week in 17 patients, and within the first 3 months in most patients. Only 15 patients continued TAT after 6 months (13%), but after one year all patients received DAT (Supplementary figure S1).

The incidence of the adverse events that occurred during follow-up are shown in table S2. Major adverse cardiac and cerebrovascular events (MACCE) occurred in 26 cases (17.7%), while clinically relevant haemorrhagic events in 21 cases (14.3%), 14 of which were major (9.5%). The incidence of all-cause death was 12.9% (19 patients); 11 subjects died from cardiovascular causes (7.4%).

### MACCE

The incidence of MACCE was numerically higher in TAT group, although not statistically significant (20.8% vs.12.5%, p = 0.266), while it was comparable between VKA and DOAC group (17% vs.18%, p = 1.000).

**Fig. 1:**
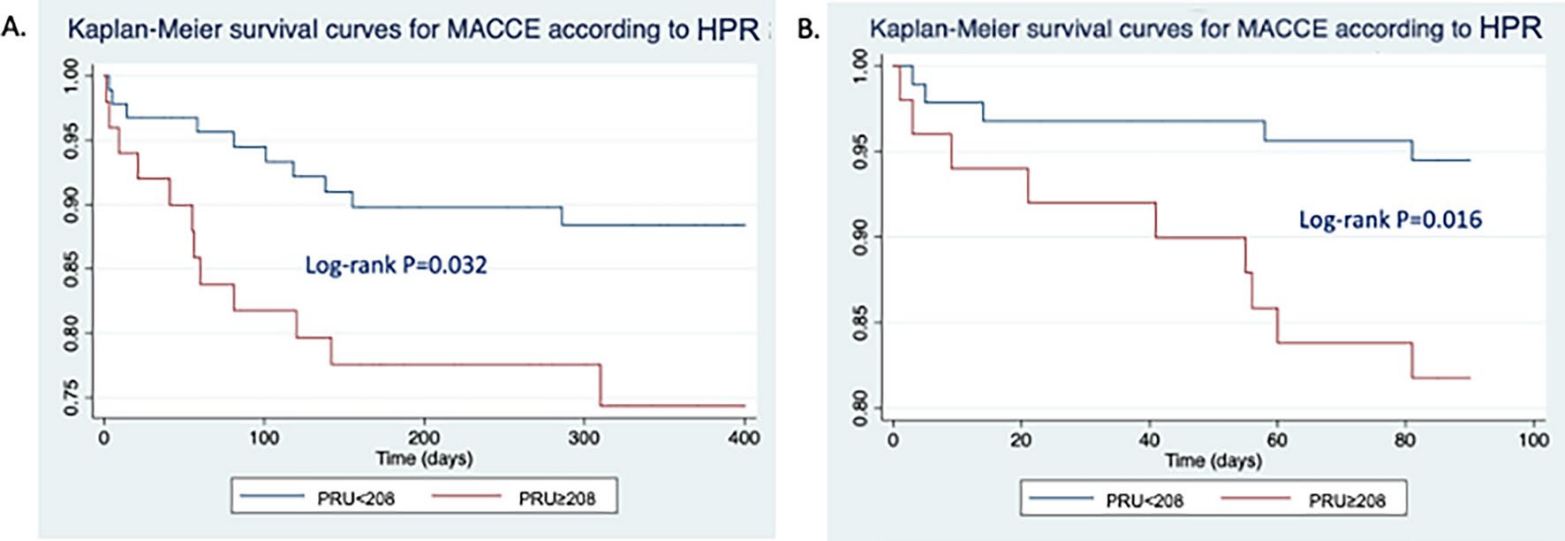
Kaplan–Meier survival curves for MACCE according to P2Y_12_ dependent HPR (cut-off 208 PRU). **A** 1-year follow-up. **B** 3-months follow-up

More than half of ischemic events occurred in the first three months of therapy (14, 56%). At 3-months follow-up, Cox regression analysis demonstrated the association between MACCE and increasing levels of PRU, together with male gender (HR for each SD variation 2.93, 95% C.I. 1.03 to 7.56, p = 0.027 and HR 0.09, 95% C.I. 0.15 to 0.53, p = 0.008, respectively; Fig. 1). The presence of CYP2C19*2 alleles also resulted to be an independent predictor of MACCE, both in the model including P2Y_12_ platelet reactivity or combined HPR. Moreover, concomitant TXA2-dependent and P2Y_12_-dependent HPR was independently associated with the incidence of MACCE (HR 19.32, 95% C.I. 2.83 to 131.95, p = 0.003, Table 2).

**Table 2:** Univariate and multivariable Cox regression analysis for the incidence of MACCE, major and non-major clinically relevant hemorrhagic events and all-cause mortality at 3-months follow-up

	MACCE				Major and non-major clinically relevant hemorrhagic events				All-cause mortality			
	Univariate		Multivariable		Univariate		Multivariable		Univariate		Multivariable	
Variable	HR (95% C.I.)	P value	HR (95% C.I.)	P value	HR (95% C.I.)	P value	HR (95% C.I.)	P value	HR (95% C.I.)	P value	HR (95% C.I.)	P value
Age*	1.29 (0.73–2.30)	0.373			1.27 (0.68–2.36)	0.452			1.93 (0.98–3.82)	0.058		
Male gender	0.34 (0.12–0.97)	0.043	0.09 (0.15–0.53)^a^ 0.07 (0.01–0.53)^b^	0.008 0.010	0.41 (0.13–1.28)	0.127			0.20 (0.06–0.63)	0.007	0.15 (0.04–0.53)	0.003
Diabetes	1.15 (0.38–3.42)	0.807			2.17 (0.70–6.73)	0.181			2.48 (0.83–7.38)	0.103		
Hypertension	0.74 (0.21–2.67)	0.654			0.60 (0.16–2.21)	0.442			2.37 (0.31–18.30)	0.405		
Dyslipidemia	0.94 (0.33–2.70)	0.907			0.72 (2.31–2.22)	0.563			2.42 (0.67–8.80)	0.179		
Active smoke	1.40 (0.31–6.26)	0.658			3.10 (0.84–11.47)	0.089			1.50 (0.33–6.77)	0.598		
Previous PCI	0.68 (0.23–2.02)	0.485			0.61 (0.18–2.03)	0.442			0.56 (0.17–1.82)	0.336		
Previous CABG	0.04 (0.00–171.60)	0.458			0.04 (0.01–334.25)	0.492			0.93 (0.12–7.13)	0.942		
Previous MI	0.93 (0.31–2.78)	0.898			0.33 (0.07–1.49)	0.149			0.51 (0.14–1.87)	0.313		
PAD	1.67 (0.51–5.48)	0.396			0.44 (0.10–2.03)	0.291			11. 28 (2.50–50.92)	0.002	19.23 (3.85–96.02)	< 0.001
Previous stroke/TIA	2.27 (0.83–6.16)	0.107			0.67 (0.86–5.17)	0.697			1.43 (0.32–6.43)	0.645		
Previous bleeding	0.43 (0.00–151.80)	0.451			0.04 (0.01–284.75)	0.484			0.90 (0.12–6.92)	0.920		
ACS	5.95 (0.78–45.46)	0.086			36.39 (0.21–6186.33)	0.170			0.53 (0.69–40.69)	0.109		
Multivessel disease	1.17 (0.37–3.72)	0.795			5.38 (0.69–41.70)	0.107			0.54 (0.18–1.61)	0.269		
LM disease	1.03 (0.32–3.28)	0.960			3.16 0.96–10.34)	0.058	3.90 (1.14–13.34)	0.030	1.60 (0.52–4.90)	0.408		
N° of stents	0.66 (0.39–1.13)	0.130			1.05 (0.67–1.65)	0.832			1.04 (0.69–1.57)	0.870		
Total stent length	0.99 (0.97–1.01)	0.173			0.99 (0.98–1.02)	0.940			1.01 (0.98–1.2)	0.901		
CHA_2_DS_2_VASc score	1.23 (0.87–1.95)	0.206			0.97 (0.61–1.55)	0.907			1.83 (1.05–3.19)	0.034		
HASBLED score	0.74 (0.33–1.67)	0.473			0.99 (0.42–2.32)	0.976			1.42 (0.88–2.30)	0.155		
White blood cell count*	1.66 (1.01–2.75)	0.047	2.66 (1.07–6.63)^b^	0.026	1.31 (0.70–2.46)	0.392			2.12 (1.28–3.50)	0.004	2.48 (1.37–4.48)	0.003
Hb	0.85 (0.64–1.46)	0.290			1.11 (0.94–1.31)	0.234			1.06 (0.86–1.32)	0.591		
Platelet*	1.22 (0.78–1.87)	0.361			1.52 (1.10–2.11)	0.011	1.66 (1.16–2.37)	0.005	1.11 (0.68–1.80)	0.675		
eGFR*	0.79 (0.45–1.36)	0.392			0.92 (0.52–1.64)	0.787			0.44 (0.23–0.82)	0.011		
INR	0.25 (0.03–2.29)	0.221			2.13 (1.08–4.20)	0.030			1.13 (0.43–2.95)	0.808		
P2Y_12_ dependent platelet reactivity [PRU] *	1.74 (1.19–2.55)	0.004	2.93 (1.03–7.56)^a^	0.027	1.18 (0.69–2.00)	0.562			0.84 (0.46–1.53)	0.574		
TXA2 dependent platelet reactivity [ARU]*	0.70 (0.47–1.04)	0.080			0.92 (0.54–1.56)	0.756			1.36 (0.71–2.59)	0.350		
Combined TXA2 and P2Y_12_ dependent HPR	3.99 (1.25–12.72)	0.019	19.32 (2.83–131.95)^b^	0.003								
CYP2C19 *2 genotype	4.03 (0.90–18.00)	0.068	5.21 (1.03–26.28)^a^ 6.16 (1.09–34.78)^b^	0.045 0.039	0.29 (0.04–2.27)	0.238			0.77 (0.16–3.64)	0.745		
DOAC vs. VKA	2.60 (0.58–11.61)	0.211			1.35 (0.37–4.99)	0.652			0.36 (0.12–1.09)	0.070	0.22 (0.07–0.74)	0.014
TAT vs. DAT	1.14 (0.38–3.40)	0.813			1.33 (0.40–4.42)	0.640			1.02 (0.33–3.13)	0.967		

*ACS* acute coronary syndrome; *CABG* coronary artery bypass grafting; *CI* confidence interval; *CVD* cardiovascular disease; *DOAC* direct oral anticoagulant; *eGFR* estimated glomerular filtration rate; *Hb* Hemoglobin; *HPR* high platelet reactivity; *HR* hazard ration; *INR* international normalized ratio; *LM* left main; *MACCE* major adverse cardiac and cerebrovascular events; *MI* myocardial infarction; *PAD* peripheral artery disease; *PCI* percutaneous coronary intervention; *TIA* transient ischemic attack; *VKA* vitamin K antagonist

*HR for each increase of 1SD

^a^Model including P2Y_12_ dependent platelet reactivity

^b^Model including combined TXA2 and P2Y_12_ dependent HPR

At 1-year follow-up, presentation as ACS and P2Y_12_ dependent platelet reactivity were independent predictors of MACCE (HR 9.91, 95% C.I. 1.33 to 73.80, p = 0.025 and HR for each SD variation 1.67, 95% C.I. 1.20 to 2.34, p = 0.003, respectively; Table 3, Fig. 1). The presence of CYP2C19*2 polymorphism was not significantly associated with the incidence of MACCE in this amount of time.

**Table 3:** Univariate and multivariable Cox regression analysis for the incidence of MACCE, major and non-major clinically relevant hemorrhagic events and all-cause mortality at 1-year follow-up

	MACCE				Major and non-major clinically relevant hemorrhagic events				All-cause mortality			
	Univariate		Multivariable		Univariate		Multivariable		Univariate		Multivariable	
Variable	HR (95% C.I.)	P value	HR (95% C.I.)	P value	HR (95% C.I.)	P value	HR (95% C.I.)	P value	HR (95% C.I.)	P value	HR (95% C.I.)	P value
Age*	0.95 (0.63–1.43)	0.800			1.39 (0.83–2.31)	0.208			2.31 (1.23–4.32)	0.009	1.98 (1.10–3.55)	0.022
Male gender	0.77 (0.32–1.83)	0.768			0.47 (0.19–1.16)	0.103			0.24 (0.09–0.66)	0.005	0.27 (0.09–0.78)	0.016
Diabetes	1.12 (0.50–2.82)	0.704			1.57 (0.63–3.90)	0.334			1.89 (0.73–4.90)	0.191		
Hypertension	0.55 (0.22–1.42)	0.221			0.57 (0.21–1.60)	0.289			3.39 (0.45–25.58)	0.236		
Dyslipidemia	1.00 (0.42–2.34)	0.997			0.61 (0.25–1.50)	0.280			2.27 (0.74–6.97)	0.151		
Active smoke	0.83 (0.19–3.55)	0.800			3.48 (1.25–9.67)	0.017			1.10 (2.52.4.82)	0.898		
Previous PCI	1.02 (0.44–2.36)	0.967			0.54 (0.21–1.44)	0.224			0.52 (0.18–1.48)	0.221		
Previous CABG	1.07 (0.25–4.57)	0.930			0.56 (0.76–4.24)	0.580			0.67 (0.09–5.06)	0.672		
Previous MI	0.96 (0.40–2.29)	0.930			0.30 (0.09–1.03)	0.057	0.27 (0.08–0.94)	0.040	0.52 (0.17–1.60)	0.254		
PAD	1.53 (0.61–3.80)	0.360			0.56 (0.18–1.69)	0.302			3.87 (1.43–10.49)	0.008	4.37 (1.55–12.34)	0.005
Previous stroke/TIA	2.27 (0.83–6.16)	0.107			0.84 (0.19–3.64)	0.816			1.66 (0.455.78)	0.425		
Previous bleeding	0.04 (0.01–29.40)	0.345			0.58 (0.08–4.20)	0.570			1.41 (0.32–6.19)	0.644		
ACS	10.0 (1.35–74.24)	0.024	9.91 (1.33–73.80)	0.025	1.84 (0.61–5.57)	0.276			2.11 (0.60–7.35)	0.240		
Multivessel disease	2.16 (0.73–6.38)	0.164			4.16 (0.96–18.01)	0.057			0.66 (0.25–1.74)	0.402		
LM disease	0.98 (0.38–2.51)	0.996			2.70 (1.07–6.80)	0.035	2.92 (1.14–7.49)	0.026	1.80 (0.69–4.75)	0.231		
N° of stents	0.82 (0.56–1.20)	0.304			1.08 (0.75–1.53)	0.681			0.88 (0.58–1.32)	0.539		
Total stent length	0.99 (0.98–1.01)	0.266			1.00 (0.99–1.02	0.728			0.99 (0.98–1.01)	0.753		
CHA_2_DS_2_VASc score	1.24 (0.90–1.72)	0.192			1.05 (0.73–1.05)	0.786			1.67 (1.14–2.43)	0.008		
HASBLED score	1.20 (0.68–2.15)	0.530			1.18 (0.60–2.31)	0.635			1.71 (0.94–3.10)	0.078		
White blood cell count*	1.36 (0.89–2.09)	0.154			1.22 (0.74–2.02)	0.442			1.67 (1.05–2.67)	0.031	1.84 (1.10–3.08)	0.020
Hb	0.67 (0.37–1.22)	0.193			1.08 (0.91–1.28)	0.401			0.99 (0.79–1.25)	0.984		
PLT*	1.07 (0.71–1.60)	0.760			1.40 (1.04–1.87)	0.027			1.06 (0.68–1.67)	0.792		
eGFR*	0.71 (0.45–1.11)	0.131			0.99 (0.63–1.55)	0.953			0.45 (0.26–0.78)	0.005		
INR	0.41 (0.10–1.79)	0.238			2.26 (1.29–3.97)	0.005			1.38 (0.70–2.75)	0.347		
P2Y_12_ dependent platelet reactivity [PRU] *	1.73 (1.26–2.46)	0.002	1.67 (1.20–2.34)	0.003	0.87 (0.54–1.39)	0.558			0.75 (0.44–1.28)	0.300		
TXA2 dependent platelet reactivity [ARU]*	0.91 (0.60–1.37)	0.649			0.82 (5.57–1.20)	0.309			1.23 (0.72–2.11)	0.448		
CYP2C19 *2 genotype	1.67 (0.56–4.98)	0.359			0.98 (0.32–3.03)	0.968			0.94 (0.26–3.41)	0.923		
DOAC vs VKA	0.91 (0.37–2.23)	0.840			1.68 (0.55–5.07)	0.355			0.38 (0.15–0.98)	0.046	0.27 (0.10–0.72)	0.009

*ACS*, acute coronary syndrome; *CABG*, coronary artery bypass grafting; *CI*, confidence interval; *CVD*, cardiovascular disease; *DOAC*, direct oral anticoagulant; *eGFR*, estimated glomerular filtration rate; *Hb*, Hemoglobin; *HR* hazard ration; *INR* international normalized ratio; *LM* left main; *MACCE*, major adverse cardiac and cerebrovascular events; MI; myocardial infarction; *PAD* peripheral artery disease; *PCI* percutaneous coronary intervention; *TIA* transient ischemic attack; *VKA* vitamin K antagonist

*HR for each increase of 1SD

A ROC curve for P2Y_12_ dependent platelet reactivity was performed in order to identify the value of PRU to be considered as a cut-off (maximum sensitivity and sensitivity for identification of patients at risk for MACCE) in the present population, in which antiplatelet and anticoagulant therapy coexist. The Area Under Curve was 0.651 (95% C.I. 0.536 to 0.817; p = 0.031) and the value identified was 186 U. Kaplan–Meier curve confirmed a significant association between MACCE and this cut-off (HR 7.61, 95% C.I. 1.70 to 34.01, p = 0.002).

We performed a separate analysis in DAT and TAT groups. Concomitant TXA2 and P2Y_12_ dependent HPR resulted to be an independent predictor of ischemic events both at 3 and 12-months follow-up in TAT group (HR 8.38, 95% C.I. 1.68–41.79, p = 0.010, and HR 6.58, 95% C.I. 1.36–31.86, p = 0.019, respectively), whereas in DAT group the same was observed for P2Y_12_ dependent platelet reactivity (HR for each SD variation 4.81, 95% C.I. 1.43–16.22, p = 0.011 and HR for each SD variation 12.20, 95% C.I. 1.33–111.78, p = 0.027, respectively). The carriage of *2 polymorphism of CYP2C19 was not significantly associated with MACCE in this sub-analysis (data not shown).

### Major and non-major clinically relevant haemorrhagic events

Major and non-major clinically relevant bleeding events were comparable between TAT and DAT group (16.5% vs. 8.9%, p = 0.225) and between DOAC and VKA group (17.0% vs. 8.5%, p = 0.212).

In the first three months after stenting, the presence of CAD involving left main (LM) coronary artery and platelets count were independently associated with the incidence of major and non-major clinically relevant haemorrhagic events (HR 3.90, 95% C.I. 1.14 to 13.34, p = 0.030 and HR for each SD variation 1.66, 95% 1.16 to 2.37, p = 0.005, respectively, Table 2).

At 1-year follow-up LM disease was confirmed an independent predictor of bleeding together with history of previous MI (HR 2.92, 95% 1.14 to 7.49, p = 0.026 and HR 0.27, 95% C.I. 0.08 to 0.94, p = 0.040, respectively). No association was found between platelet reactivity and bleeding (Table 3).

### All-cause mortality

The rate of all-cause mortality was not different between patient in TAT and those in DAT (12.1% vs. 14.2%, p = 0.801), while a higher risk of death was seen in patients on VKA (21.2% vs 9.0%, p = 0.038).

The independent predictors at 12-months of follow-up were: age (HR for each SD variation 1.98, 95% C.I. 1.10 to 3.55; p = 0.022), male gender (HR 0.27, 95% C.I. 0.09 to 0.78, p = 0.016), the presence of PAD (HR 4.37, 95% C.I. 1.55 to 12.34, p = 0.005), white blood cells count (HR for each SD variation 1.84, 95% C.I. 1.10 to 3.08; p = 0.020) and therapy with DOAC (HR 0.27, 95% C.I. 0.10 to 0.72, p = 0.009, Table 3). In the first three months all were confirmed except from age (Table 2).

## Discussion

The present prospective registry was meant to evaluate the clinical impact of HPR and genetic polymorphism associated with clopidogrel resistance in high-risk patients discharged with antiplatelet and anticoagulant therapies in combination. The main result of the present study is that P2Y_12_ dependent HPR was independently associated with increased ischemic events at both 3-month and 1-year follow-up. The presence of CYP2C19*2 alleles resulted an independent predictor of MACCE at 3 months but not at 1 year of follow-up. No association was found between platelet reactivity and bleeding risk, or all-cause mortality.

### Antithrombotic pattern and ischemic risk

In a real-world unselected population, the choice of the more appropriate antithrombotic pattern was various, as expected, but TAT was still the most prescribed and it was carried on for at least one month after discharge in most subjects. The decision on the type of antithrombotic pattern in favor of TAT was mainly driven by coronary complexity, while CHA_2_DS_2_-VASc e HAS-BLED score did not differ between TAT or DAT group. In TAT and DAT clopidogrel was far the preferred antiplatelet agent, as expected.

Despite the established association between antithrombotic combination therapy and bleeding risk, our data showed that the incidence of ischemic events was at least comparable to that of bleeding ones. Unexpectedly, the results of the present analysis highlight the need of not undervaluing thrombotic risk, especially early after stenting. Actually, most of these events occurred in the first three months after enrolment, even if the risk persisted across the subsequent follow-up. It is worth considering that this was a very high-risk population: mean age was 78 years old, nearly one subject out of two had undergone a coronary revascularization before index hospitalization, which occurred for ACS in 70% of cases. However, these reasons underlie the strength of our registry, since it represents a picture of a real-world unselected population, not necessary resembling that enrolled in RCT, but often encountered in clinical practice. Moreover, these data confirm that even if bleeding is the most feared event, elderly carries a significant high ischemic risk, as already demonstrated [26, 27], and therefore adequate antithrombotic therapy should not be precluded in older patients for age-related reasons only.

In contrast to what expected, neither the type of antithrombotic pattern or of anticoagulant agent were associated with a statistical different incidence of ischemic or bleeding events. This may be interpreted as a consequence of the non-randomized nature of this registry and of the limited sample size. Interestingly, we confirmed the survival benefit of DOAC as compared with VKA, as already observed in other observational registries [28–31].

### Role of PFT

The use of PFTs to tailor antiplatelet drugs in cardiovascular patients has been debated for the last ten years. In spite of numerous studies and meta-analyses confirming the associations between HPR and cardiovascular outcomes among patients treated with P2Y_12_ inhibitors [21, 32, 33], RCT failed to demonstrate any benefit of a PFT-guided strategy compared to standard care [34–36]. More recently, Galli et al. demonstrated in a meta-analysis of data from 20,743 patients that the use of platelet function or genetic testing to guide the selection of antiplatelet therapy in patients undergoing PCI reduced the risk of cardiovascular death, MI, stent thrombosis, stroke, without any trade-off in safety [37].

In the contest of combined antithrombotic therapy, the evidence on PFT application is still limited. Some recently published registry enrolling patients with ACS or PCI and an indication for oral anticoagulant failed to demonstrate any significant relationship between HPR and MACCE or between low platelet reactivity and bleeding [38, 39].

In our registry, HPR was a potent independent predictor of ischemic events, especially in the first three months: patients with PRU levels above the cut-off of 208 at Verify-now assay showed a significantly higher risk of MACCE, as already demonstrated in ACS patient in DAPT [40, 41]. The prevalence of HPR was consistent with other aforementioned trials and the first published study assessing this issue (n = 50, 34%) [42]. Since the 208 cut-off was validated in patients in DAPT, we hypothesized that in this setting, where antiplatelets are always combined with anticoagulant agents and aspirin is omitted in several cases, a different value could better discriminate subjects at higher ischemic risk. Indeed, the cut-off of 186 U was more effective, suggesting that when DAPT is early discontinued, a deeper P2Y_12_ inhibition may be required, despite anticoagulation background. Besides, dual HPR (both to aspirin and clopidogrel) was associated with extremely high risk of MACCE. This condition, already described in other contests [42, 43], characterized patient whose platelets are functionally not inhibited at all after coronary stenting, justifying the particularly high ischemic risk. This issue deserves particular mention considering the latest shortening of TAT to 1 week as the recommended default strategy in this setting [14, 44], therefore enhancing the risk of ischemic events in patients who respond poorly to clopidogrel [1].

Moreover, our data demonstrate that the presence of CYP2C19*2 allele confers a higher risk in the first months after the acute events, and HPR by ADP also later on. These data are in line with those obtained previously by our group and others [45, 46]. On-treatment HPR is a complex phenomenon for which pharmacogenetic accounts only for a part. Genotyping patients for CYP2C19*2 allele allows the identification of patients who immediately need an alternative drug to clopidogrel. Phenotyping patients for the entity of platelet inhibition provides information about a factor which maintain its prognostic role until 12 months after the acute event. Besides, as concerned the subgroup analysis by antithrombotic pattern, the association between functional HPR and MACCE was confirmed in both TAT and DAT group, whereas genetic testing did not reach the statistical significance threshold. However, we cannot exclude that this result could be partially attributed to the limited sample size, considering the missing values of this test.

We believe that these data demonstrate the pivotal role PFT (both functional and genetic) in guiding antithrombotic strategy in the setting of combined antithrombotic therapy, since we are often dealing with ACS patients receiving mostly clopidogrel alone or DAPT for a brief amount of time.

### Limitations

Several limitations of this analysis should be acknowledged. Firstly, non-randomized design may have produced selection bias; however, this registry was born to evaluate a real-world population as discharged from hospital. Secondly, the sample size is limited. Nevertheless, since the latest guidelines recommendations release, antithrombotic pattern has become more homogeneous, making impossible to perform any comparison between TAT and DAT. Finally genetic testing was possible only in a subset of subjects and did not include the search for *3 allele.

## Conclusions

The present analysis underlines the clinical impact of HPR on ischemic events in a high-risk real-word population of patients treated with either DAT or TAT. That of patients with ACS or PCI with concomitant AF represents an ideal field for the application of a tailored use of antithrombotic therapy, since different type of drugs and duration of therapy can be combined in a myriad of possible strategies that need to be personalized according to patient’s profile. The opportunity to add laboratory markers of drug response to classical risk scores could be of help in clinical decision-making.

## Supplementary Information

Below is the link to the electronic supplementary material.

Supplementary file1 (DOCX 34 KB)
