# Supplementary material for: Clinical impact of high platelet reactivity in patients with atrial fibrillation and concomitant percutaneous coronary intervention on dual or triple antithrombotic therapy
Source: J Thromb Thrombolysis. 2023 Mar 11;55(4):667–79. doi: 10.1007/s11239-023-02784-z (PMC10147742; doi:10.1007/s11239-023-02784-z)
Supplement: Supplementary file 1 — Supplementary file1 (DOCX 34 KB) [file 11239_2023_2784_MOESM1_ESM.docx]

**Supplementary materials.**

**Table S1.** Baseline characteristics of the study population according to antithrombotic pattern and type of anticoagulant.

|  | **TAT** | | | **DAT** | | |
| --- | --- | --- | --- | --- | --- | --- |
|  | **VKA**  **(n=38)** | **DOAC**  **(n=53)** | **P value** | **VKA**  **(n=9)** | **DOAC**  **(n=47)** | **P value** |
| **Age** [yrs], (mean±SD) | 74±9 | 78±6 | 0.011 | 80±8 | 79±7 | 0.521 |
| **Male sex,** n (%) | 28 (73.7) | 34 (64.2) | 0.336 | 6 (66.7) | 31 (66) | 0.967 |
| **Hypertension,** n (%) | 32 (84.2) | 42 (79.2) | 0.542 | 9 (100) | 39 (83) | 0.181 |
| **Dyslipidemia**, n (%) | 27 (71.1) | 31 (58.5) | 0.219 | 5 (55.6) | 23 (48.9) | 0.716 |
| **Smokers**, n (%)  Former Smokers, n (%) | 5 (13.2)  18 (47.4) | 6 (11.3)  21 (39.6) | 0.663 | 1 (11.1)  3 (33.3) | 4 (8.5)  16 (34) | 0.969 |
| **Diabetes mellitus**, n (%) | 18 (47.4) | 16 (30.2) | 0.095 | 2 (22.2) | 14 (29.8) | 0.645 |
| **Family history of CVD**, n (%) | 6 (15.8) | 9 (17) | 0.880 | 0 | 7 (14.9) | 0.216 |
| **BMI** (mean±SD) | 25.7±3.6 | 26.7±3.5 | 0.240 | 27.3±3.5 | 28.1±4.7 | 0.633 |
| **Prior MI**, n (%) | 12 (31.6) | 21 (39.6) | 0.431 | 2 (22.2) | 18 (38.3) | 0.356 |
| **Prior PCI**, n (%) | 16 (42.1) | 25 (47.2) | 0.632 | 2 (22.2) | 21 (44.7) | 0.210 |
| **Prior CABG**, n (%) | 5 (13.2) | 3 (5.7) | 0.213 | 1 (11.1) | 3 (6.4) | 0.614 |
| **Prior TIA/stroke,** n (%) | 9 (23.7) | 2 (3.8) | 0.004 | 2 (22.2) | 5 (10.6) | 0.336 |
| **PAD,** n (%) | 14 (36.8) | 14 (26.4) | 0.413 | 3 (33.3) | 17 (36.2) | 0.723 |
| **Prior bleeding,** n (%) | 0 | 5 (9.4) | 0.051 | 1 (11.1) | 6 (12.8) | 0.891 |
| **ACS,** n (%) | 31 (81.6) | 37 (69.8) | 0.203 | 5 (55.6) | 30 (63.8) | 0.639 |
| **UA,** n (%) | 8 (21.1) | 14 (26.4) | 0.043 | 1 (11.1) | 4 (8.5) | 0.166 |
| **NSTEMI,** n (%) | 13 (34.2) | 20 (37.7) |  | 1 (11.1) | 21 (44.7) |  |
| **STEMI,** n (%) | 10 (26.3) | 3 (5.7) |  | 3 (33.3) | 5 (10.6) |  |
| **LVEF** [%], (mean ± SD) | 45±11 | 45±11 | 0.770 | 44±12 | 49±11 | 0.193 |
| **N of diseased vessels 1** n (%)  **2** n (%)  **3** n (%) | 2 (2-3)  8 (21.1)  16 (42.1)  14 (36.8) | 2 (2-3)  9 (17)  22 (41.5)  21 (39.6) | 0.653 | 1 (1-2)  6 (66.7)  2 (22.2)  1 (11.1) | 1 (1-2)  22 (46.8)  15 (31.9)  10 (21.3) | 0.279 |
| **LM disease, n (%)** | 16 (43.2) | 16 (30.2) | 0.203 | 3 (33.3) | 6 (12.8) | 0.124 |
| **N of treated vessels,** median (IQR) | 2 (1-2) | 1 (1-2) | 0.563 | 1 (1-1) | 1 (1-2) | 0.211 |
| **N stent** median (IQR) | 2 (1-3) | 2 (1-3) | 0.944 | 1 (1-2) | 1 (1-2) | 0.933 |
| **Total stent length,** mm (mean±SD) | 52±30 | 54±37 | 0.810 | 47±29 | 40±33 | 0.599 |
| **CHA_2_DS_2_-VASc score** (mean±SD) | 5.0±1.6 | 4.7±1.2 | 0.263 | 5.1±1.1 | 4.6±1.2 | 0.277 |
| **HAS-BLED score** (mean±SD) | 2.8±0.8 | 2.1±0.5 | 0.010 | 2.4±0.5 | 2.3±0.6 | 0.469 |
| **Statin,** n (%)  **Atorvastatin,** n (%)  **Rosuvastatin,** n (%)  **Pravastatin,** n (%)  **Simvastatin,** n (%) | 33 (86.8)  33 (86.8)  0  0  0 | 49 (92.4)  37 (69.8)  9 (17)  0  3 (5.7) | 0.483 | 6 (66.7)  5 (55.6)  0  0  1 (11.1) | 40 (85.1)  27 (57.4)  1 (2.1)  7 (14.9)  5 (10.6) | 0.338 |
| **PPI,** n (%)  **Lansoprazol,** n (%) **Omeprazol,** n (%) **Pantoprazol,** n (%) | 34 (89.5)  7 (18.4)  2 (5.3)  25 (65.8) | 51 (96.2)  7 (13.2)  0  44 (83) | 0.231  0.134 | 8 (88.9)  1 (11.1)  0  7 (77.8) | 43 (91.5)  10 (21.3)  1 (2.1)  32 (68.1) | 0.999  0.862 |
| **WBC** [x10^3/^uL] (mean±SD) | 8.66±3.55 | 7.50±3.04 | 0.099 | 7.47±1.64 | 7.86±2.56 | 0.661 |
| **Hb** [g/dL], (mean±SD) | 11.4±2.0 | 12.1±1.8 | 0.097 | 13.7±7.0 | 12.0±2.0 | 0.490 |
| **Platelets** [/mL], (mean±SD) | 222447± 71673 | 219641± 97551 | 0.881 | 220222± 108587 | 222638± 77530 | 0.936 |
| **MCV** (mean±SD) | 89.3±12.1 | 89.0±6.1 | 0.877 | 90.6±3.0 | 89.7±6.8 | 0.701 |
| **Creatinin** [mg/dL], (mean±SD) | 2.2±1.6 | 1.1±0.4 | <0.001 | 1.1±0.3 | 1.1±0.3 | 0.585 |
| **eGFR** [ml/min], (mean±SD) | 41±27 | 53±20 | 0.017 | 51±19 | 58±23 | 0.349 |
| **Dialysis,** n (%) | 7 (18.4) | 0 | 0.001 | 0 | 0 | - |

Abbreviations: BMI, body mass index; CABG, coronary artery bypass grafting; CVC, cardiovascular disease; DOAC, direct oral anticoagulant; DTA, dual antithrombotic therapy; eGFR, estimated glomerular filtration rate; Hb, Hemoglobin; IQR, interquartile range; LVEF, left ventricular ejection fraction; LM, left main; MCV, mean corpuscular volume; MI; myocardial infarction; NSTEMI, non-ST elevation myocardial infarction; PCI, percutaneous coronary intervention; PPI, proton pump inhibitor; SD, standard deviation; STEMI, ST elevation myocardial infarction; TAT, triple antithrombotic therapy; TIA, transient ischemic attack; UA, unstable angina; VKA, vitamin K antagonist; WBC, white blood cell count.

**Figure S1.** Antithrombotic pattern during follow-up.

Abbreviations: DAT: dual antithrombotic therapy; TAT, triple antithrombotic therapy.

**Table S2.** Adverse events according to antithrombotic therapy pattern.

|  | **TAT** | | **DAT** | |
| --- | --- | --- | --- | --- |
|  | **VKA**  **(n=38)** | **DOAC**  **(n=53)** | **VKA**  **(n=9)** | **DOAC**  **(n=47)** |
| **MACCE** | | | | |
| ACS, n (%) | 4 (10.5) | 7 (13.2) |  | 3 (6.4) |
| Critical limb ischemia, n (%) | 1 (2.6) |  |  |  |
| Mesenteric ischemia, n (%) |  | 1 (1.9) |  | 1 (2.1) |
| Ischemic stroke, n (%) | 2 (5.3) | 1 (1.9) |  |  |
| Fatal MACCE, n (%) | 1 (2.6) | 2 (3.8) |  | 3 (6.4) |
| **Bleeding events** | | | | |
| **Major** |  |  |  |  |
| ICH, n (%) |  | 3 (5.7) |  | 1 (2.1) |
| Fatal ICH, n (%) |  | 1 (1.9) |  |  |
| GI bleeding, n (%) |  | 3 (5.7) | 2 (22.2) | 2 (4.3) |
| GU bleeding, n (%) | 1 (2.6) |  |  |  |
| Procedural, n (%) |  | 1 (1.9) |  |  |
| **Non-major clinically relevant,** n (%) | 1 (2.6) | 5 (9.4) |  | 1 (2.1) |
| **Minor,** n (%) | 1 (2.6) | 1 (1.9) |  | 2 (4.3) |
| **Death from any cause,** n (%) | 7 (18.4) | 4 (7.5) | 3 (33.3) | 5 (10.6) |
| **Death from cardiovascular cause,** n (%) | 5 (13.2) | 3 (5.7) | 1 (11.1) | 3 (6.4) |

Abbreviations: ACS, acute coronary syndrome; DAT, dual antithrombotic therapy; DOAC, direct oral anticoagulant, GI, gastrointestinal, GU, genitourinary; ICH, intracranial hemorrhage; MACCE, major adverse cardiac and cerebrovascular events; TAT, triple antithrombotic therapy, VKA, vitamin K antagonist.
